# Reply to Timonin and Cooley: Varied effects of counterfactuals in 42 countries underscore value of within-country counterfactual

**DOI:** 10.1073/pnas.2407918121

**Published:** 2024-06-03

**Authors:** Leah R. Abrams, Mikko Myrskylä, Neil K. Mehta

**Affiliations:** ^a^Department of Community Health, Tufts University, Medford, MA 02155; ^b^Max Planck Institute for Demographic Research, Rostock 18057, Germany; ^c^Population Research Unit, University of Helsinki, Helsinki 00014, Finland; ^d^Max Planck–University of Helsinki Center for Social Inequalities in Population Health, Rostock 18057, Germany; ^e^Department of Epidemiology, University of Texas Medical Branch, Galveston, TX 77555

Timonin and Cooley (1) demonstrate the importance of baseline mortality conditions in counterfactual analyses aimed at understanding life expectancy trends. We are delighted that our original analysis “The ‘double jeopardy’ of midlife and old age mortality trends in the United States” (2) has inspired further demographic studies exploring mortality dynamics.

Our paper addressed the question of how different age groups contributed to the stagnating trend in US life expectancy after 2010. Timonin and Cooley asked how other countries’ life expectancies would have changed if they experienced post-2010 mortality trends observed in the United States. Their analysis is a useful reminder of a mathematical property of life table calculations: initial levels of mortality and relative change in mortality interact to produce changes in life expectancy. An important detail of the Timonin and Cooley calculation is that they explore relative, not absolute, change in mortality. Our analysis (2) used a linear extrapolation of the average annual change in death rates in the preperiod, in which case baseline mortality levels are less consequential.

The results of Timonin and Cooley’s counterfactual analysis (1), and those of an earlier letter by Polizzi and Dowd (3), highlight the importance of choosing an appropriate counterfactual in demographic studies of mortality. We argue that the most reasonable counterfactual to identify the age-specific contributions to stagnating US life expectancy is the United States’ own earlier mortality conditions. Often, a counterfactual is based on a different country’s mortality experience (3). Such a counterfactual, however, is less relevant to understanding a country’s recent and future life expectancy path than the counterfactual of a country’s own past, which embodies the pre-existing set of risk factors and demographic conditions operating in that population.

When Timonin and Cooley (1) apply the US relative change to the baseline mortality rates of 42 other countries, they observe quite varied effects on life expectancy, especially among men. Polizzi and Dowd (3) also found that conclusions about the relative role of midlife and old-age mortality in the United States varied depending on the counterfactual country used. This variation further underscores the value of using a within-country counterfactual to understand the sources of life expectancy trends in the United States and to provide a realistic portrait of what life expectancy improvements the United States may achieve (or fail to achieve).
